# Validation of automated surveillance of healthcare-associated infections using electronic screening algorithms

**DOI:** 10.1017/ash.2023.375

**Published:** 2023-09-29

**Authors:** Hyunju Lee, Seunghee Ryu, Hyejin Yang, Jeongyoung Lee, Soyeon Park, Eun Ok Kim, Jiwon Jung, Sung-Han Kima

## Abstract

**Background:** Surveillance of healthcare-associated infection (HAI) is the basis of infection prevention programs. However, manual review of medical records is a labor-intensive and time-consuming process. We evaluated the diagnostic performance of automated surveillance of HAI using electronic screening algorithms. **Methods:** Between April and June 2022, we conducted surveillance of HAI manually and automatically using electronic screening algorithm on 75 units (general medical and surgical wards and ICUs) in a 2,700-bed, tertiary-care hospital in South Korea. Algorithms for surveillance of HAI were developed accordance with NHSN surveillance definitions (Fig. 1). Catheter-associated urinary tract infections (CAUTIs) were automatically detected when eligible pathogen and fever (>38°C) were matched within infection window period. Other specific types of infection were automatically classified based on laboratory results that met NHSN criteria. After the algorithm showed possible cases that met laboratory-confirmed bloodstream infection (LCBI) criteria, we excluded secondary BSIs using the automatic surveillance algorithm. We analyzed sensitivity, specificity, positive predictive value (PPV), and negative predictive value (NPV) for the automated surveillance system compared to manual surveillance. **Results:** An algorithm for detecting CAUTI showed 98.7% sensitivity (78 of 79), 100.0% specificity (2,443 of 2,443), 100.0% PPV (78 of 78), and 100.0% NPV (2,443 of 2,444). For CLABSI, the algorithm had 97.3% sensitivity (214 of 220), 98.3% specificity (5,759 of 5,861), 67.7% PPV (214 of 316), and 99.9% NPV (5,759 of 5,765). In total, 102 cases of possible CLABSI were identified by the algorithm, and 76 (74.5%) were eventually diagnosed as secondary BSIs. Also, by chart review, 20 BSIs (19.6%) were present on arrival in ER (ER-POA). In 4 cases (3.9%), an original pathogen reoccurred in a repeated infection timeframe (RIT), and 2 cases (2%) were mucosal barrier injury-LCBI (MBI-LCBI). When we additionally performed manual surveillance for intra-abdominal infection secondary BSI, ER-POA, and assigning pathogen to original BSI in RIT, PPV increased to 87.7% (214 of 244). **Conclusions:** Algorithm for automated surveillance of CAUTI had good performance; however, automated surveillance of CLABSI was suboptimal. More elaborate screening algorithm for diagnosis CLABSI is needed, and further studies are needed to determine whether an automated surveillance system can reduce workload for surveillance of HAI.

**Figure:**
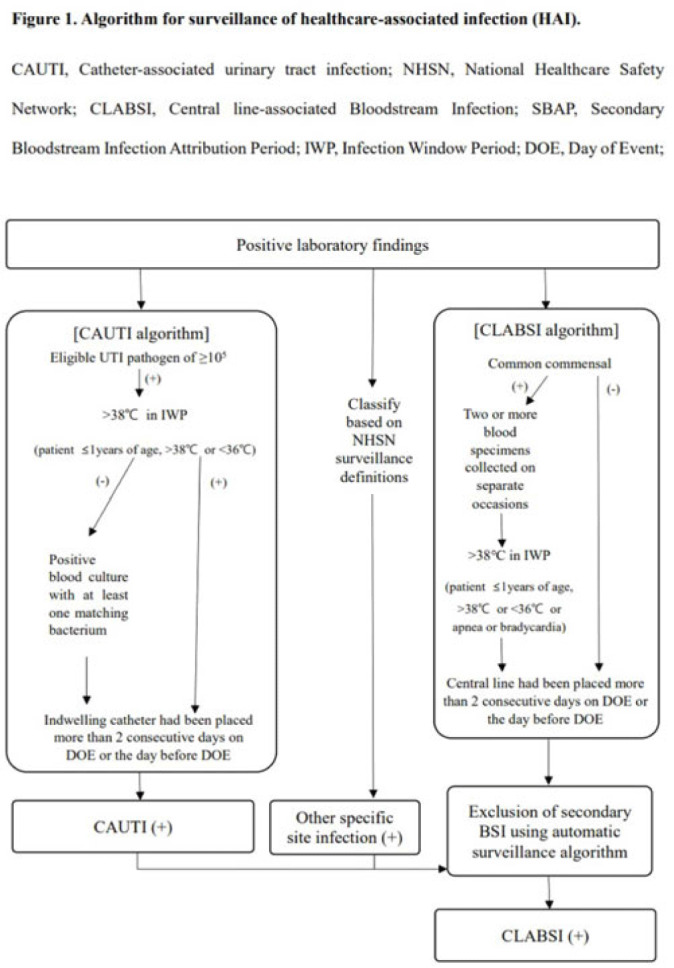

**Figure:**
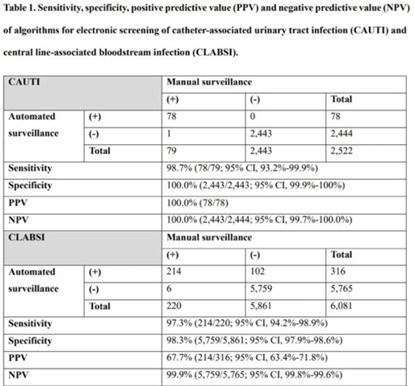

**Disclosures:** None

